# How gender shapes public speaking in virtual reality: a multimodal analysis

**DOI:** 10.3389/frai.2026.1954571

**Published:** 2026-10-16

**Authors:** Marion Ristorcelli, Rémy Casanova, Magalie Ochs

**Affiliations:** 1 Laboratoire d'Informatique et des Systémes, Aix-Marseille University, Marseille, France; 2 Institut des Sciences du Mouvement, Aix-Marseille University, Marseille, France

**Keywords:** gender differences, multimodal non-verbal behavior, public speaking performance, virtual audience, virtual reality

## Abstract

Virtual Reality (VR) has proven effective for public speaking training, particularly due to its ability to simulate immersive speaking environments and automatically extract behavioral cues reflecting performance. However, limited attention has been paid to the impact of speaker gender and the gendered appearance of the virtual audience on the public speaking experience in VR. To address this gap, we conducted an experiment with 55 participants who performed oral presentations within a VR simulation that allowed for the systematic manipulation of the virtual audience's gendered appearance and social attitude. We captured a comprehensive dataset of multimodal nonverbal behavioral data, alongside subjective evaluations from both the participants and an expert using standardized and non-standardized questionnaires. By combining statistical analysis and machine learning approaches, our findings revealed no significant effect of speaker gender on her/his emotional experience or her/his public speaking performance. In contrast, male and female speakers exhibited distinct nonverbal behavioral patterns, identified through both expert evaluation and automatic multimodal behavioral analysis. Moreover, Virtual audience social attitude influenced participants' emotional experience and self-reported performance, whereas no significant effect of audience gendered appearance was detected. These findings highlight the importance of considering behavioral variability when studying and assessing public speaking in VR.

## 1 Introduction

Proficiency in oral communication is a cornerstone of professional success and employability. Effective speaking skills are crucial for the various stages of a career, including job interviews, recruitment processes, professional advancement, and promotions within business environments (Syathroh, 2020; Chan, 2011; Lim et al., 2026). However, public speaking competencies, characterized by confidence and persuasion, are traditionally attributed to men (Koenig et al., 2011). This sociocultural association can create an unfavorable environment for female speakers, potentially triggering stereotype threat. Such pressures can impose an additional psychological constraint in high-stakes speaking situations, modulating the speaker's emotional experience and performance (Kleinlogel et al., 2021; Pardal et al., 2020). In the public speaking literature, emotional experience is typically assessed through participants' self-reported anxiety levels (Ul Huda et al., 2024) or broader emotional states (Stevanovic et al., 2019). In contrast, public speaking performance is generally evaluated either through self-reported performance assessments (Ul Huda et al., 2024) or by external raters (Zhou et al., 2021), with judgments based on a combination of verbal cues (e.g., speech rate, use of filler words, and pause duration) and nonverbal cues (e.g., gaze direction, facial expressions, and open gestures) displayed during the speech (Notaro et al., 2021). Indeed, several works indicate that female speakers tend to experience higher levels of public speaking anxiety and are more likely to fear being perceived as poor speakers than male speakers (Perveen et al., 2018; Rahman Hz, 2022). The literature also largely reports that higher self-reported public speaking anxiety is tied to shorter speech duration and poorer self-perceived speech quality (Gallego et al., 2022). More broadly, previous studies have reported gender differences in verbal and nonverbal behavior during public speaking (Leaper and Ayres, 2007; Newman et al., 2016; Singhal et al., 2018; Leung et al., 2018; Latu et al., 2019). However, these studies have predominantly emphasized verbal behaviors, whereas nonverbal behavior has often been investigated using only a limited set of cues. Consequently, it remains unclear whether male and female speakers exhibit genuine multimodal nonverbal behavioral differences, and whether such differences are associated with performance or subjective emotional experience. To address this question, the present study focuses specifically on multimodal nonverbal behavioral cues.

In this regard, Virtual Reality (VR) has emerged as a promising public speaking training tool that allows for the collection of a speaker's verbal and nonverbal behavior (Chollet et al., 2015; Palmas et al., 2019; Jakubowski et al., 2019; Palmas et al., 2021; Ochs et al., 2024). By recording these objective multimodal cues, VR provides a suitable environment to observe whether actual behavioral differences exist between male and female speakers, offering a foundation for the future development of personalized performance feedback to enhance speaking skills. Beyond behavioral measurement, VR is also widely utilized for its capacity to reduce the anxiety associated with public speaking (Daniels et al., 2020; Valls-Ratés et al., 2022, 2023; Schmidt-Peter et al., 2026). By providing immersive, controllable, and repeatable speaking scenarios, it enables individuals to practice in realistic yet safe environments. Nevertheless, few systems take into account the environmental context, beyond the social attitudes of the virtual audience, and its influence on participants' emotional experience (Pertaub et al., 2002). Among the environmental characteristics that may shape this experience, the composition of the virtual audience, particularly the gendered appearance of its members, has received relatively little attention. Some existing virtual audiences consider only virtual characters with male appearance (Pertaub et al., 2002; Chollet et al., 2015), others consider a few virtual characters with female appearance in their virtual audience (Biesmans et al., 2020; Mostajeran et al., 2020; Palmas et al., 2019, 2021), and relatively few include an equal number of virtual characters with male and female appearance (Glémarec et al., 2022, 2021; Bettahi et al., 2026). The choice of the gendered appearance of virtual characters is not neutral, as virtual characters are perceived differently depending on their appearance (Armando et al., 2022). While a character with a male appearance is often associated with power (Nunamaker et al., 2011), expertise (Nunamaker et al., 2011), and knowledge (Baylor and Kim, 2004), a character with a female appearance is more likely to be perceived as likable (Nunamaker et al., 2011) or attractive (Lunardo et al., 2016). These stereotypes, which persist even in human-machine interactions, can influence not only the learners' performance but also their self-perception and self-efficacy (Armando et al., 2022). In the context of VR public speaking, the role of virtual audience gendered appearance remains poorly understood. A pioneering study in this field reported that female speakers experienced higher levels of anxiety than male speakers when presenting in front of a virtual audience composed only of character with male appearance (Pertaub et al., 2002). Although these findings suggest that virtual audience composition may influence speakers' emotional experience, they do not disentangle the respective contributions of the speaker gender and the audience gendered appearance. Indeed, the observed differences could simply reflect the well-established tendency for female speakers to report higher levels of public speaking anxiety than men, independently of audience composition. Consequently, it remains unclear whether the gendered appearance of the virtual audience, the gender of the speaker, or the interaction between the two drives these differences.

To fill these gaps, we investigate the respective influence of speaker gender and virtual audience gendered appearance on public speaking in VR. Specifically, we examine how these factors shape the speaker's emotional experience and their performance, assessed through both self-reported performance and expert-rated performance, as well as through the analysis of their multimodal behavior. To this end, this work explores the following research questions:

1. Does speaker gender or the gendered appearance of the virtual audience influence emotional experience and performance during a VR public speaking task?
2. Does speaker gender or the gendered appearance of the virtual audience influence speakers' multimodal behavior during a VR public speaking task?

To answer these research questions, this study employs a multimodal analysis to investigate how gender shapes public speaking in VR. One original aspect of our approach is the consideration of two kinds of measures: (1) *subjective* measures reflecting emotional experience and performance from both the users' and the expert's perspectives, and (2) *objective* behavioral cues exhibited by users during the public speaking task. Furthermore, to investigate the impact of gendered appearance, the proposed methodological approach combines traditional statistical analysis with machine learning methods. Beyond traditional statistics, the machine learning approach provides deeper insights by ranking the most influential behavioral cues and revealing the distinct behavioral patterns that differentiate male and female speakers during the task.

The remainder of this paper is organized as follows. Section 2 reviews the related work to establish the theoretical framework and identify gaps in current research. Section 3 details the experimental setup and the VR environment, while Section 4 describes the two complementary data analysis approaches used to address our research questions: statistical analysis and machine learning. Section 5 examines the influence of gender on emotional experience and performance through statistical analyses. It also investigates objective behavioral cues using a machine learning approach to uncover emergent gender-based patterns during the public speaking task. Section 6 discusses these findings in the context of existing literature and presents the study limitations and directions for future research. Finally, Section 7 concludes the paper.

## 2 Related work

### 2.1 Gender influence in public speaking situations

A growing body of literature has investigated the role of gender differences across a number of psychological traits that may influence performance and behavior during public speaking tasks (Babcock et al., 2017; Azmat and Petrongolo, 2014; Niederle, 2016). These studies have reported that females are more risk-averse, less competitive, and possess lower levels of self-confidence and self-esteem. Furthermore, they tend to suffer more under pressure and when receiving negative feedback than male speakers (De Paola et al., 2021). In addition, several studies have observed that female speakers tend to report higher levels of public speaking anxiety than male speakers in self-report questionnaires (Rodero and Larrea, 2022; De Paola et al., 2021; Broeckelman-Post and Pyle, 2017; Boĺıvar-Cruz and Verano-Tacoronte, 2018). However, this subjective experience is not always mirrored by physiological responses, with some studies finding no significant difference or even higher anxiety levels in male speakers (Rodero and Larrea, 2022), suggesting a complex decoupling between subjective states and biological markers of stress. This discrepancy extends to the perception of competence. Research indicates that female speakers frequently report lower levels of self-perceived communication competence, a gap that can persist even after formal training in communication skills (Broeckelman-Post and Pyle, 2017). However, this self-perception often diverges from expert-rated performance. For instance, evaluations by third-party instructors have shown no significant gender differences in the quality of presentations delivered by students (Broeckelman-Post et al., 2020). This suggests that the gender gap in public speaking may be driven more by gendered expectations and internalized stereotypes, which influence how speakers perceive their own efficacy, than by actual disparities in skill (Broeckelman-Post et al., 2020). Nevertheless, the role of the evaluator cannot be ignored, as external raters may also be influenced by implicit gender stereotypes, potentially biasing their evaluation of speakers' performance.

These societal expectations not only shape perception but may also manifest in distinct behavioral patterns. Communication styles are often guided by gendered norms, where men are associated with assertiveness and competitiveness, while women are linked to supportive and helpful roles. These norms translate into observable behavioral differences. Female speakers tend to exhibit more expressive facial expressions, higher voice pitches, and more frequent smiling and eye contact (Delbosc et al., 2024; Singhal et al., 2018; Blasberg et al., 2023). Conversely, male speakers are more likely to adopt open postures than female speakers (Latu et al., 2019; Newman et al., 2016). Several studies have also highlighted physiological differences in speech production. In particular, the average fundamental frequency of the voice ranges from approximately 80 to 180 Hz in men and from 125 to 300 Hz in women (Pisanski et al., 2021). Beyond pitch, vocal intonation, which relies on variations in fundamental frequency, plays a central role in expressiveness, charisma, the emotional dimension of speech, and audience engagement (Breitenstein et al., 2001; Yang, 2024; Hincks, 2005). As a result, some verbal features commonly associated with effective public speaking may partly reflect physiological sex differences in addition to behavioral adaptations, making the interpretation of gender differences more complex. Despite these insights, empirical research specifically focusing on the objective behavioral differences between male and female speakers during public speaking remains scarce. Moreover, although verbal features have received considerable attention, nonverbal communication, which constitutes a substantial part of a speaker's delivery, remains comparatively overlooked (Singhal et al., 2018). Taken together, these observations highlight the need for a more comprehensive investigation of nonverbal behavior during public speaking. Focusing on multimodal nonverbal behavioral cues provides an opportunity to better characterize gender-related behavioral differences beyond the limited set of cues examined in previous studies. Furthermore, to the best of our knowledge, these behavioral disparities have never been explored within the controlled environment of VR, despite the increasing use of these tools for training.

To achieve a holistic understanding of how gender shapes public speaking within VR, it is essential to disentangle the subjective self-reported experiences of users from the perspectives of external experts, while simultaneously bridging the gap in objective data regarding multimodal nonverbal behavior in these immersive environments.

### 2.2 Virtual reality for public speaking: audience influence and behavioral analysis

VR provides immersive and controllable environments that can simulate real-world social situations while maintaining strict experimental control, making it a valuable platform for studying human behavior in public speaking contexts (Zhang et al., 2026). In the literature, VR systems for public speaking are primarily deployed for two complementary objectives: as a therapeutic tool for Virtual Reality Exposure Therapy (VRET) to reduce public speaking anxiety (Schmidt-Peter et al., 2026), and as a pedagogical tool to train oral competence and improve overall performance (Zhang et al., 2026).

Regarding the management of public speaking anxiety, a central focus of these simulations is the design of the virtual audience, as its characteristics directly shape the speaker's emotional experience. Research has extensively explored how varying the audience size, the visual realism of characters, and their behaviors influence the speaker (Schmidt-Peter et al., 2026). Among these, the social attitude of the audience, expressed through nonverbal cues such as posture, gaze direction, and facial expressions, has emerged as a critical variable whose influence is well-documented. While early behavioral models defined these attitudes through simple valence and arousal values (Chollet and Scherer, 2017), a more recent framework has introduced nuanced dimensions such as affective stance, epistemic stance, and engagement (Ristorcelli et al., 2024). The combination of these dimensions, translated into specific nonverbal behaviors, allows for the representation of a wide spectrum of social attitudes, including positive and negative ones. For instance, a virtual audience exhibiting a negative attitude elicits significantly higher anxiety in the speaker than one with a positive attitude (Pertaub et al., 2002). Interestingly, while this pioneering study also noted differences in anxiety levels between male and female speakers when facing a male audience, the gender of the audience has remained largely unexplored in subsequent research. In the present study, we aim to fill this gap. We employ the behavioral model of social attitudes defined by Ristorcelli et al. (2024) to simulate positive and negative social attitudes and modulate the emotional experience, while simultaneously investigating the specific and often overlooked influence of virtual audience gender.

Parallel to anxiety reduction, VR is widely used to enhance public speaking skills through the analysis of multimodal behavior. The core of these systems lies in providing speakers with feedback, either in real time or a posteriori, to offer insights into the quality of their delivery (Palmas et al., 2019; Jakubowski et al., 2019). To provide such feedback, VR systems utilize their ability to capture multimodal behavioral cues. Performance evaluation in VR is grounded in social sciences and humanities literature, relying on a combination of verbal cues (e.g., speech rate, use of filler words, and pause duration) and nonverbal cues (e.g., gaze direction, facial expressions, and open gestures) (Ristorcelli et al., 2025). Recent advancements have integrated supervised machine learning to establish a link between these automatically extracted multimodal cues and subjective ratings provided by external raters (Azaïs et al., 2015; Ristorcelli and Ochs, 2026). By doing so, researchers can identify which specific objective cues, such as loudness or gaze direction (Ristorcelli and Ochs, 2026), most strongly correlate with high-performance perception.

Our work adopts a similar methodological framework. However, rather than predicting performance, we use these same nonverbal behavioral cues, typically used to assess public speaking quality, to investigate whether gender differences in public speaking can be objectively detected and how these cues relate to performance.

## 3 Materials and methods

### 3.1 Virtual reality experimental setup

The Revitalize Revitalizetool (Ochs et al., 2024) was employed in this study to create a controlled public speaking environment within a laboratory setting. Developed using the Unity game engine and deployed on a wireless Meta Quest Pro VR headset, the system is built around two core capabilities: first, the ability to simulate complex public speaking scenarios by manipulating audience characteristics such as social attitude, size, and gendered appearance; and second, the ability to extract and analyze multimodal behavioral data from the speaker.

To leverage the simulation capabilities of the tool, we designed a scenario specifically tailored to investigate the influence of virtual audience gender, operationalized through the gendered appearance of the virtual characters. We implemented an audience configuration consisting of four virtual characters arranged in a semicircle (Figure 1). This spatial arrangement ensured that all characters were at an equal distance from the speaker, providing equivalent visual and perceptual conditions and allowing the speaker to perceive the entire virtual audience within a single horizontal field of view. This specific number of characters also facilitated the controlled manipulation of the audience's gendered appearance. Accordingly, the virtual audience could consist entirely of female-appearing characters, entirely of male-appearing characters, or of a mixture of both. In the mixed-gender condition, two characters were randomly selected from the pool of four male-appearing and four female-appearing virtual characters. To further modulate the participants' emotional experience and introduce varying levels of difficulty, we also manipulated the social attitude of the virtual audience, a factor widely recognized as having a significant impact on speaker anxiety. Following the behavioral models developed by Ristorcelli et al. (2024), we simulated two distinct conditions: positive and negative attitudes. In the positive condition, the virtual audience displayed agreement with the speaker, attentiveness, and happiness. Conversely, in the negative condition, the virtual audience expressed disagreement, distraction, and unhappiness. To ensure consistency within each scenario, the entire virtual audience adopted a single attitude, with each character randomly selecting animations from a predefined set corresponding to the experimental condition.

**Figure 1:**
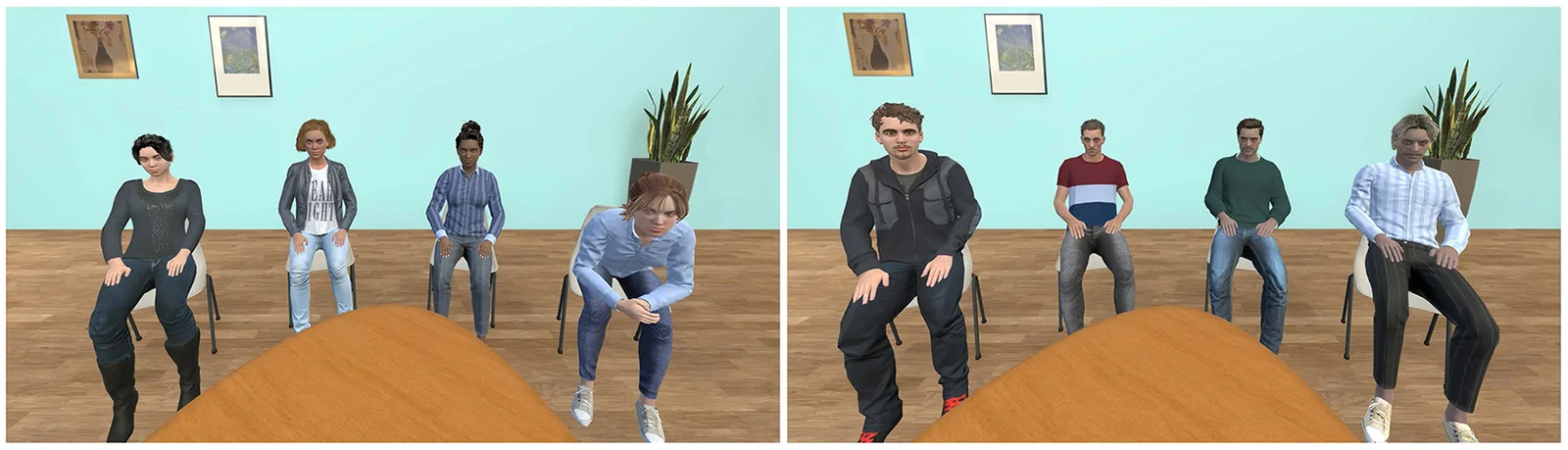
Examples of the virtual audience gendered-appearance conditions used in the experiment. Left: an audience composed of four female-appearing characters. Right: an audience composed of four male-appearing characters. Mixed-gender audience consisted of two female-appearing and two male-appearing characters, randomly selected from pools of four female-appearing and four male-appearing characters, respectively. The positions of the virtual characters within the audience were randomly assigned for each experimental session.

Parallel to these simulations, the system's data extraction capabilities were used to capture the speakers' behavior. Audio recordings and nonverbal tracking cues were captured directly via a wireless Meta Quest Pro headset, used without controllers. The Meta XR Interaction SDK was leveraged to enable the capture of head and hand positions, gaze direction, and facial muscle activations derived from FACS blendshapes. Additionally, an external camera was placed directly in front of the speaker to record the sessions for later expert evaluation. A set of multimodal behavioral features was extracted from the raw signals and audio recordings. These features, previously established by Ristorcelli et al. (2025) to assess public speaking performance, were repurposed in this study to investigate gender differences in behavioral patterns. However, while both types of features were available, the current analysis focuses exclusively on nonverbal cues to provide a comprehensive characterization of gender differences in multimodal nonverbal behavior during public speaking, an aspect that remains comparatively underexplored in the literature.

### 3.2 Participants

A total of 55 participants (29 males, 26 females), aged 18 to 61 years (*M* = 31.35, *SD* = 13.15) were recruited through a dual campaign involving posters across several university campuses and social media outreach. The study protocol was approved by the Ethics Committee of Aix-Marseille University (file no. 2024-09-12-10). All participants met the following inclusion criteria: (1) being an adult and a native French speaker; (2) no history of psychiatric disorders; (3) the ability to use a VR headset without glasses to ensure accurate facial expression recognition; and (4) a Personal Report of Public Speaking Anxiety (PRPSA) score not exceeding the sub-clinical threshold of 134 (Elsey et al., 2020).

### 3.3 Experimental protocol

A few days before the experiment, participants were informed about the study objectives and instructed to prepare six short presentations in advance using personal notes. They received a list of presentation topics covering various themes, including a personal introduction, a description of their hometown or city of residence, a summary of a book, movie, or series, a passion or hobby, a life project, and a description of a typical day. During the experiment, a different topic was randomly assigned to each of the six presentations to ensure variety and avoid repetition.

Upon arrival at the laboratory, participants were informed about the experimental procedure and provided written informed consent. They then completed a pre-experiment questionnaire to assess their baseline emotional state and initial level of public speaking anxiety. Participants were also asked to indicate how they had prepared for the presentations prior to the experiment by selecting one of the following categories: no prior rehearsal, mental rehearsal, or oral rehearsal aloud in front of one or more people. The experimental session consisted of six oral presentations, corresponding to the combination of three audience gender modalities (male, female, and mixed) and two audience attitudes (positive and negative), delivered in a VR environment using a Meta Quest Pro headset. The order of these six experimental conditions was randomized for each participant. Each presentation followed the same standardized procedure. Before each trial, participants were given 2 min to review their notes before standing on a designated marker facing the external camera. After calibration of the VR headset and verification of the system, the experimenter started the external video recording and launched the Unity-based virtual environment. Once immersed, participants faced the virtual audience assigned to the current experimental condition. When they indicated they were ready, the experimenter initiated the multimodal data recording, capturing both verbal and nonverbal behaviors, and simultaneously triggered the audience animations corresponding to the selected condition. Each presentation lasted approximately 3 min. At the end of each presentation, participants were instructed to clap their hands to facilitate synchronization between the VR audio recordings and the external video during post-processing. The experimenter then stopped both the multimodal recording and the VR simulation. Participants subsequently completed post-trial questionnaires assessing their self-reported performance and emotional experience, as described in Section 3.4.1. This sequence was repeated for all six experimental conditions. After completing all presentations, participants received a gift voucher as compensation for their participation. The experimental session, including the pre-experiment questionnaires and the six oral presentations, lasted approximately 90 min.

Following data collection, all recorded presentations were evaluated by an expert using a dedicated Unity application allowing replay of the experimental sessions. To minimize potential bias and prevent direct comparisons between multiple presentations from the same participant, recordings were presented to the expert in a randomized order. The expert assessed both the overall presentation quality and specific verbal and nonverbal behaviors according to the subjective measures described in Section 3.4.1.

### 3.4 Evaluation framework

This section describes the framework used to evaluate the impact of the experimental conditions on the speaker. The assessments are divided into two categories based on the data source: human-based subjective judgments and system-based objective measures. Subjective assessments include participants' self-reports (emotional experience and self-reported performance) as well as expert-rated performance and behavioral evaluations provided by a public speaking expert. Conversely, objective assessments include all features automatically extracted from the VR headset, specifically the multimodal nonverbal behavior measured during the experiment.

#### 3.4.1 Subjective measures

Regarding participants' self-reports, we distinguished between two categories of self-reported measures: emotional experience and self-reported performance. To assess the emotional experience, participants completed the Self-Assessment Manikin (SAM) and the Personal Report of Confidence as a Speaker (PRCS) following each presentation. The SAM is a standardized nonverbal instrument that directly measures affective responses (Bradley and Lang, 1994) and is widely used to evaluate subjective emotional states in experimental settings (Gil, 2009). It assesses three dimensions: valence, arousal, and dominance, each rated on a 5-point scale. In the present study, emotional state was assessed using the valence dimension of the SAM, following the approach adopted by Girondini et al. (2024). Higher valence scores indicate a more positive emotional state. Additionally, participants completed the PRCS to measure public speaking anxiety. We employed the validated French version proposed by Heeren et al. (2013), consisting of 12 items rated on a 5-point Likert scale. The final PRCS score was calculated as the average of these items, where a higher score indicates greater public speaking anxiety. Finally, participants rated their overall performance on a 7-point Likert scale, where higher scores indicated better self-reported performance.

Regarding the external subjective evaluation, we asked an experienced public speaking expert, with several years of experience coaching speakers for public scientific events and performing professionally, to evaluate the participants' public speaking performances. Two complementary assessments were conducted. First, the expert provided an expert-rated performance score using a 7-point Likert scale. Second, the expert rated specific aspects of the speaker's verbal and nonverbal behavior using an assessment grid comprising 20 items organized into five behavioral dimensions, with each item rated on a 5-point Likert scale:

- **Verbal behavior (7 items):** assessed time management, hesitations, intonation, speech rate, loudness, vocabulary richness, and language register. For example, the expert evaluated whether the speaker used language appropriate to the semi-formal context while avoiding colloquial or inappropriate expressions.
- **Gestures (5 items):** assessed the potential for improvement, staticity, agitation levels, the alignment between gestures and speech, and body openness. For example, the expert evaluated whether the use of the arms and hands was consistent with the discourse.
- **Posture (3 items):** assessed the potential for improvement, body protection, and muscular tension. For instance, the expert rated the speaker's level of body protection, ranging from an awkward posture to a relaxed and at-ease posture.
- **Body movements (3 items):** assessed the potential for improvement, movement staticity, and agitation. For example, the expert evaluated the extent to which the speaker's movements in space reflected agitation.
- **Gaze direction (2 items):** assessed the potential for improvement and the balance of gaze distribution toward the virtual audience. For example, the expert evaluated whether the speaker distributed their gaze evenly across the audience.

This evaluation grid was co-designed with the expert to reflect the behavioral dimensions routinely considered during public speaking assessments while also aligning with the behavioral features automatically extracted from the recordings. This correspondence was intended to facilitate subsequent analyses comparing objective behavioral measurements with subjective expert judgments. Although the automatic analyses presented in this study focus exclusively on nonverbal behavioral features, the expert assessment intentionally retained both verbal and nonverbal dimensions. In practice, evaluating a public speaking performance independently of its verbal delivery would be artificial, as verbal and nonverbal behaviors are naturally integrated in expert judgment. Retaining the verbal dimension also allows us to explore potential gender differences in expert-rated performance. To ensure a reliable and comprehensive assessment, the expert reviewed each recorded session using a dedicated Unity-based application. As illustrated in Figure 2, the application provided synchronized multimodal views of each performance, allowing the expert to inspect verbal and nonverbal behaviors from complementary perspectives before completing the assessment.

**Figure 2:**
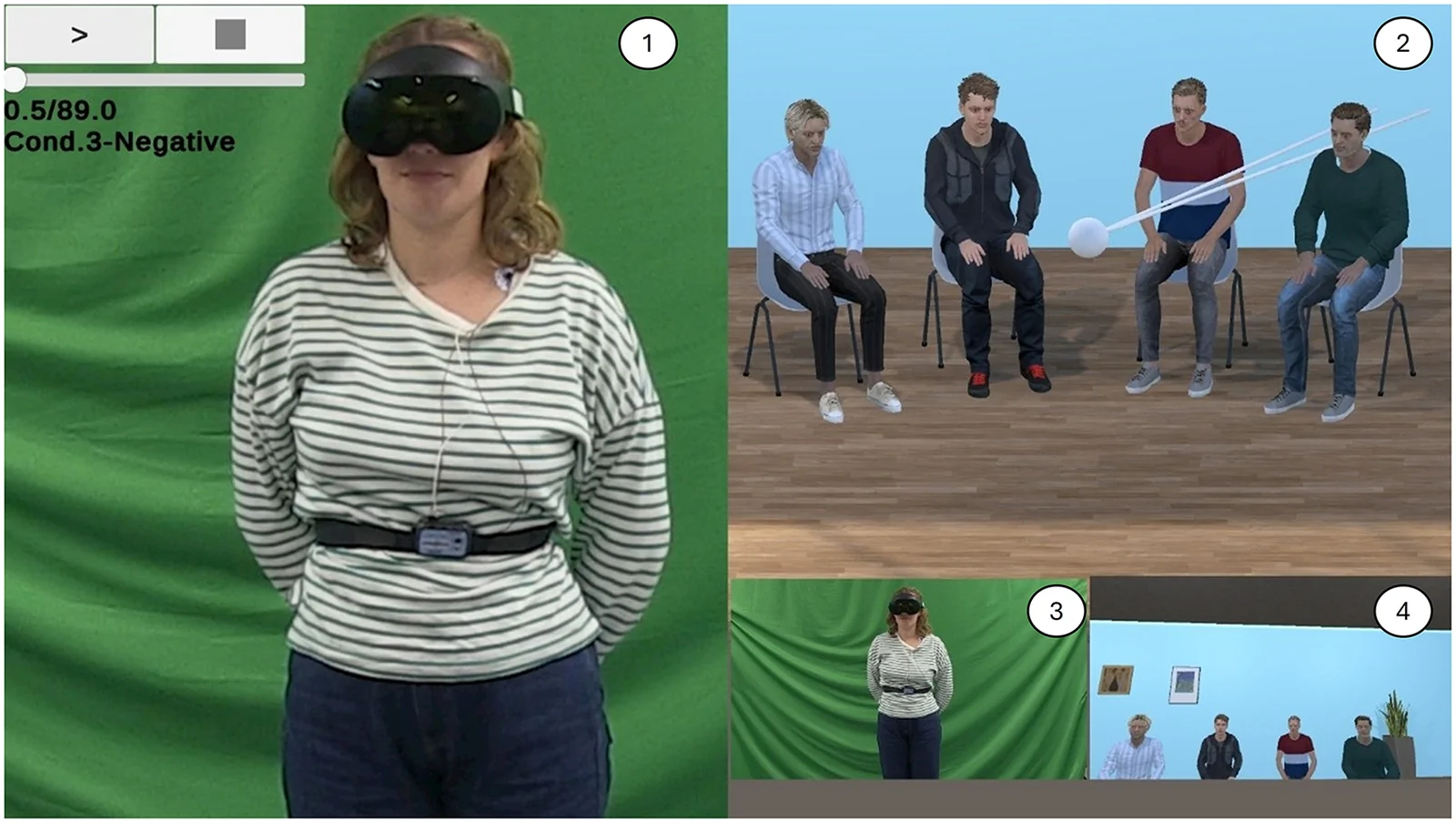
Expert evaluation interface used to assess public speaking performance. The interface provides four synchronized views of the participant during the speech: **(1)** a zoomed-in external view, **(2)** a top-down view of the virtual audience with real-time gaze visualization, **(3)** a wide-angle external view of the participant, and **(4)** a first-person VR view. These complementary views enabled the expert to simultaneously assess verbal and nonverbal behaviors throughout the public speaking session.

#### 3.4.2 Objective measures

Parallel to the subjective assessments, objective behavioral measures were automatically extracted from the raw data collected by the Meta Quest Pro VR headset. Following the methodology introduced by Ristorcelli et al. (2025), we extracted a set of interpretable multimodal nonverbal behavioral features from the recorded signals. Accordingly, 33 automatically extracted multimodal nonverbal behavioral features were retained to investigate whether female and male speakers exhibit distinct behavioral patterns and whether these cues alone are sufficiently informative to distinguish between speaker genders. The extracted features were organized into four complementary categories:

- **Gaze features (6 features):** included the gaze diversity score based on the proportion of virtual audience members viewed, the median and interquartile range of successive gaze durations, the proportion of time spent looking at the audience, the proportion of time spent looking away toward the floor or ceiling, and gaze-direction entropy.
- **Body movement features (3 features):** included horizontal displacement amplitude, total head distance traveled per unit of time, and body-movement entropy.
- **Facial expression features (11 features):** included the occurrence frequencies of ten facial blendshapes (Lip Corner Puller, Cheek Raiser, Brow Lowerer, Inner Brow Raiser, Lid Tightener, Lip Corner Depressor, Lip Stretcher, Outer Brow Raiser, Upper Lid Raiser, and Lip Tightener) as well as facial expression entropy.
- **Gesture and posture features (13 features):** included the proportion of hand detection; the frequencies of upward, downward, and neutral palm orientations; the frequencies of open, closed, and neutral hand configurations; the proportion of open posture; the median and interquartile range of successive palm-up gesture durations; the total distance traveled by the hands per unit of time; hand-opening entropy; and palm-orientation entropy.

## 4 Data analysis methods

To investigate how gender influences the public speaking experience in VR, two complementary analysis methods were employed. Statistical analyses were first conducted to examine the effects of audience social attitude, audience gendered appearance, and speaker gender on participants' emotional experience, self-reported performance, expert-rated performance, and expert behavioral evaluations. In addition, a machine learning approach was used to determine whether the automatically extracted behavioral features contained sufficient information to discriminate speaker gender and to identify the behavioral patterns associated with each gender. Accordingly, this approach aimed to identify the features contributing most to gender discrimination by accounting for the combined effects and interactions between multiple nonverbal cues.

### 4.1 Statistical analysis

We conducted statistical analyses to assess the extent to which the audience social attitude (positive or negative), the audience gender composition (male, female, mixed), and the speaker gender (male or female) influenced the speakers' emotional experience, their self-reported performance, expert-rated performance, and the expert's behavioral evaluations. Prior to these analyses, baseline emotional state and baseline public speaking anxiety, collected before exposure to the VR environment, were compared between male and female speakers using the Mann–Whitney *U* test. The type of preparation undertaken before the experiment was also compared between male and female speakers using Pearson's chi-squared test of independence. The dependent variables collected during the experiment, measured using Likert scales, showed distributions that deviated from normality. Therefore, we used the Aligned Rank Transform (ART) ANOVA, a non-parametric approach to analysis of variance designed for ordinal data. Accordingly, three-factor mixed-effects models were used to examine the effects of speaker gender, audience gender composition, social attitude, and their interactions on the dependent variables. Speaker gender was treated as a between-subject factor, whereas audience gender composition and social attitude were treated as within-subject factors. To account for the repeated-measures structure of the design, subject ID was included as a random intercept in all ART models. Statistical significance was assessed using Type III F tests with Kenward–Roger adjusted degrees of freedom.

Finally, whenever appropriate, post hoc analyses were performed using Aligned Rank Transform Contrasts (ART-C). For significant effects, descriptive statistics are reported as medians and interquartile ranges (IQRs).

### 4.2 Machine learning-based analysis

Beyond expert-rated performance, we sought to determine whether gender differences in oral performance are supported by objectively measured nonverbal behavioral patterns. To this end, we complemented the statistical analyses with a machine learning approach. Unlike conventional statistical analyses, which examine the effects of individual variables, this approach evaluates whether multimodal nonverbal behavioral features, considered jointly, contain sufficient information to discriminate between female and male speakers. It also identifies the behavioral features that contribute most to this discrimination, thereby providing insight into the nonverbal cues associated with female and male speakers. Comparing these objective behavioral markers with expert-rated performance further makes it possible to examine the correspondence between objectively measured behavioral patterns and subjective expert judgments.

To address this complementary question, speaker gender prediction was formulated as a supervised binary classification task based exclusively on automatically extracted nonverbal behavioral features. A Multilayer Perceptron (MLP) classifier was trained using these features to predict speaker gender. Our dataset comprised 330 presentations corresponding to 55 participants, each delivering six presentations. To increase the number of samples while preserving the temporal structure of each speech, each presentation was divided into three equal-duration segments corresponding to the beginning, middle, and end of the speech, following the methodology proposed by Biancardi et al. (2025). This resulted in a dataset of 990 observations, each corresponding to a speech segment derived from the original presentations. Data were first split using a rigorous stratification procedure on the target variable into a training set and an independent test set. In particular, we ensured that subjects included in the training set did not appear in the test set. The resulting training and test sets exhibited a balanced gender distribution, as reported in Table 1. Hyperparameter optimization was then performed on the training set using a random search over 10 hyperparameter configurations. For each configuration, Recursive Feature Elimination (RFE) combined with k-fold cross-validation (*k* = 5) was used to identify the subset of behavioral features that maximized model performance. The final model was then retrained using the optimal hyperparameters and selected feature subset before being evaluated on the independent test set. In accordance with the evaluation framework described by Ristorcelli and Ochs (2026), we assessed model performance using the weighted F1-score and the Area Under the Receiver Operating Characteristic Curve (ROC-AUC). Finally, SHapley Additive exPlanations (SHAP) (Lundberg and Lee, 2017) were used to interpret the predictions of the final model by quantifying the contribution of each behavioral feature while accounting for the joint influence of all features. This explainability analysis complements the classification results by identifying the nonverbal behaviors that contribute most to discriminating between female and male speakers. By revealing which objectively measured behaviors drive the model's decisions, it provides an interpretable characterization of the behavioral patterns associated with speaker gender. These patterns can then be compared with expert-rated performance to determine whether gender differences are supported by measurable behavioral cues.

**Table 1:** Gender distribution of the dataset observations after splitting into the training set (used for hyperparameter optimization and feature selection) and the independent test set (used for the final evaluation).

Dataset	Male speakers (%)	Female speakers (%)	Total (%)
Training set	414 (53.5%)	360 (46.5%)	774 (78.2%)
Test set	108 (50.0%)	108 (50.0%)	216 (21.8%)

Values are reported as the number of observations and the corresponding percentages(%).

## 5 Results

### 5.1 Baseline comparisons

Before examining the effects of the experimental conditions, we first compared baseline emotional state, public speaking anxiety, and the preparation method used before the experiment between male and female speakers. These measures were collected before exposure to the VR environment and therefore reflect initial differences rather than effects of the experimental 1conditions.

There was no significant association between speaker gender and the type of training performed before the task, χ^2^(2) = 2.32, *p* = 0.31. No significant difference between male and female speakers was observed for baseline emotional state assessed with the SAM valence score (*U* = 387, *p* = 0.88) or for baseline public speaking anxiety assessed with the PRCS (*U* = 459.5, *p* = 0.17).

We next examined whether gender differences emerged in participants' emotional experience during the VR public speaking task despite the absence of baseline differences.

### 5.2 Influence of experimental conditions on user experience

This section addresses the first research question, focusing on whether *the gender of the speaker or the gendered appearance of the virtual audience influences the emotional experience and performance during a VR public speaking task*, while also considering the impact of the virtual audience social attitude. As described in Section 3.4.1, emotional experience was assessed using self-reported measures of public speaking anxiety (PRCS) and emotional state (SAM), whereas performance was evaluated through both the participants' self-reported performance and expert-rated performance. Overall, participants successfully completed the public speaking task, with presentations lasting an average of 2.53 ± 0.98 min (mean ± SD), indicating sustained engagement throughout the task.

#### 5.2.1 Emotional experience

Regarding public speaking anxiety, the analysis of PRCS scores revealed *a significant main effect of audience social attitude*, *F*(1, 265) = 13.20, *p* < 0.001. However, no significant effects were observed for audience gendered appearance, *F*(2, 265) = 0.30, *p* = 0.74, or speaker gender, *F*(1, 53) = 1.26, *p* = 0.27, nor were any interaction effects significant. Specifically, as reported in Figure 3A, participants reported significantly lower public speaking anxiety when facing a positive audience (*median* = 1.75, *Q*_1_ = 1.25, *Q*_3_ = 2.17) than when facing a negative audience (*median* = 1.92, *Q*_1_ = 1.33, *Q*_3_ = 2.42), although anxiety scores remained generally low across conditions, with median values below the midpoint of the 5-point scale.

**Figure 3:**
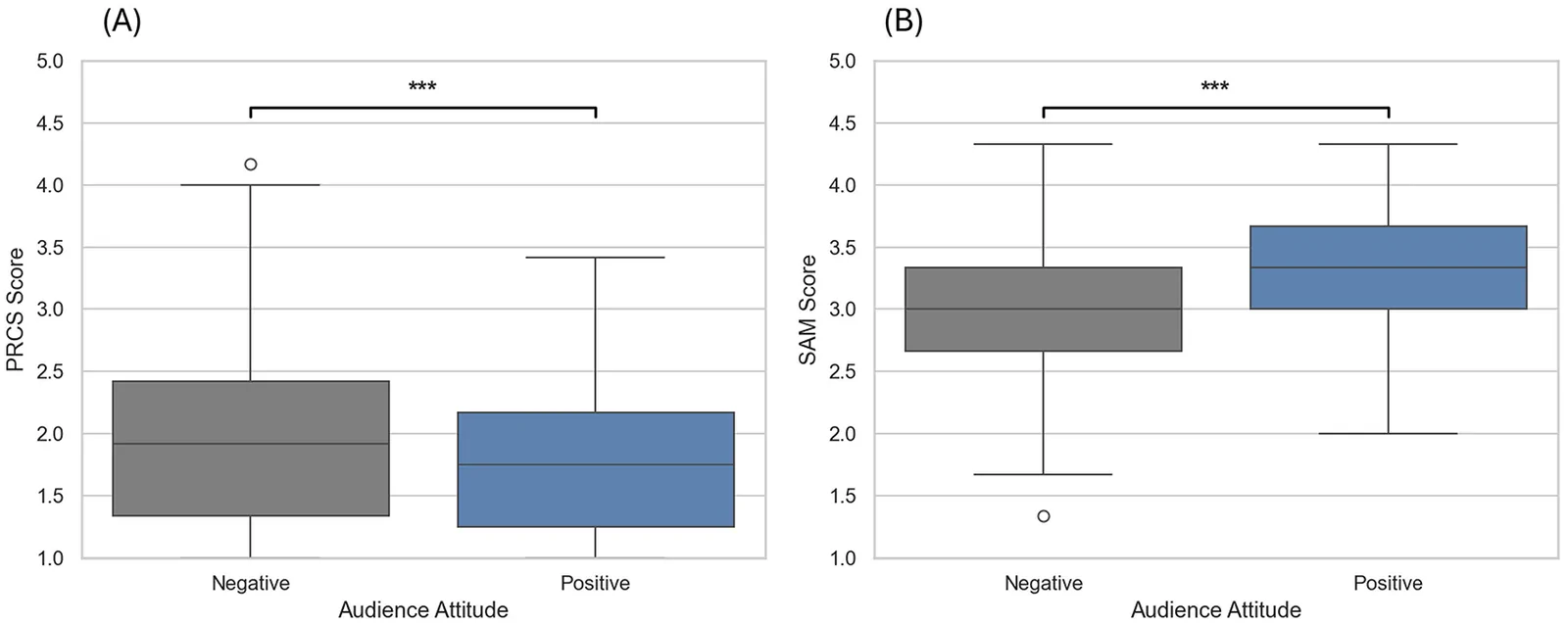
Effect of audience attitude (positive vs. negative) on emotional experience: **(A)** public speaking anxiety (PRCS) and **(B)** emotional state (SAM). Boxes represent the interquartile range (IQR) and the central line indicates the median. *p < 0.05, **p < 0.01, ***p < 0.001.

Regarding emotional state, SAM valence scores revealed a *significant main effect of virtual audience social attitude*, *F*(1, 265) = 55.57, *p* < 0.001. As reported in Figure 3B, participants reported more positive emotions when presenting to a positive audience (*median* = 3.33, *Q*_1_ = 3.00, *Q*_3_ = 3.67) than to a negative one (*median* = 3.00, *Q*_1_ = 2.67, *Q*_3_ = 3.33).

In summary, these results indicate that the virtual audience social attitude strongly influences participants' emotional experience, as *participants reported lower anxiety and more positive emotions when facing a positive audience*. No significant effect of speaker gender or audience gendered appearance was observed on either anxiety or emotional state. Having established the effect of audience attitude on participants' emotional experience, we now examine whether these differences translate into differences in self-reported performance and expert-rated performance.

#### 5.2.2 Self-reported and expert-rated performance

Regarding self-reported performance, the analysis revealed a *significant main effect of virtual audience social attitude*, *F*(1, 265) = 16.94, *p* < 0.001. However, no significant effects were observed for audience gendered appearance, *F*(2, 265) = 1.53, *p* = 0.22, or speaker gender, *F*(1, 53) = 1.34, *p* = 0.25, nor were any interaction effects found between these factors. Virtual audience attitude had therefore a significant impact on self-reported performance independently of audience gendered appearance and speaker gender. More specifically, participants reported slightly higher self-reported performance scores after presenting to a positive audience (*median* = 5, *Q*_1_ = 4, *Q*_3_ = 6) than after presenting to a negative one (*median* = 5, *Q*_1_ = 3, *Q*_3_ = 5) as indicated in Figure 4. Regarding expert-rated performance, no significant effects were found for audience attitude, *F*(1, 265) = 1.57, *p* = 0.21, audience gendered appearance, *F*(2, 265) = 0.84, *p* = 0.43, or speaker gender, *F*(1, 53) = 0.73, *p* = 0.40. These findings indicate that expert-rated performance did not differ according to audience attitude, audience gendered appearance, or speaker gender.

**Figure 4:**
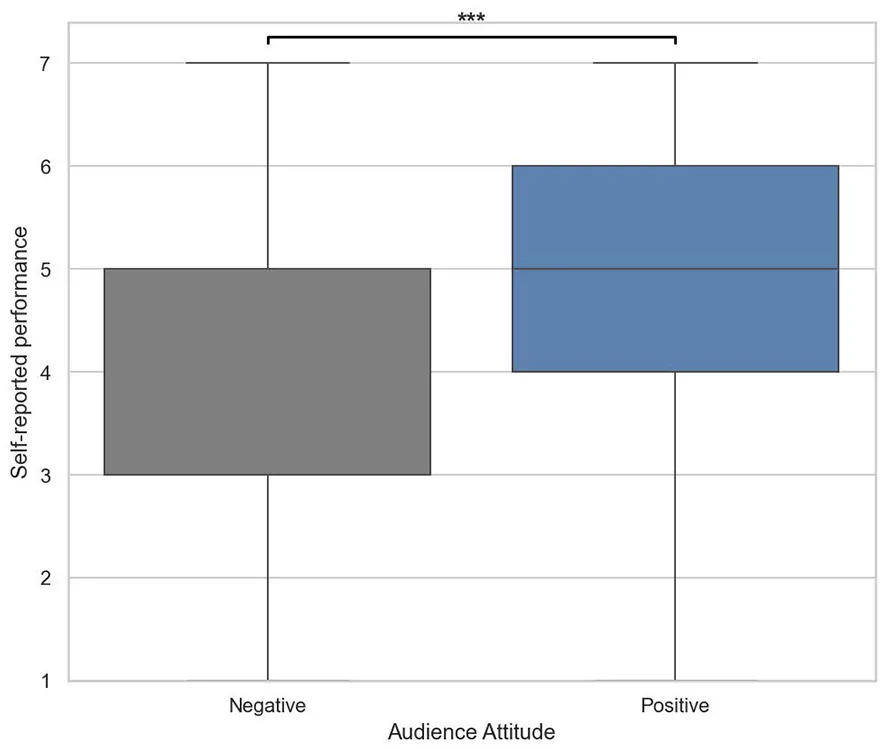
Effect of audience attitude (positive vs. negative) on self-reported performance. Boxes represent the interquartile range (IQR) and the central line indicates the median.*p < 0.05, **p < 0.01, ***p < 0.001.

Consistent with the previous findings, participants' self-reported performance was influenced by the virtual audience social attitude. Interestingly, a discrepancy emerged: *while participants perceived their performance to be better when facing a positive audience, expert-rated performance showed no significant differences*. In the next section, we examine whether male and female speakers nevertheless exhibit distinct multimodal nonverbal behavioral patterns, despite comparable expert-rated performance.

### 5.3 Gender differences in multimodal nonverbal behavior

This section addresses the second research question, focusing on whether *the gender of the speaker or the virtual audience influences speakers' multimodal behavior during a VR public speaking task*, while also accounting for the impact of the virtual audience social attitude. Although gender had no significant impact on either self-reported performance or expert-rated performance, the following behavioral analysis offers a deeper perspective on the influence of gender during the public speaking task. The proposed analysis first examines whether expert behavioral evaluations revealed gender differences. Subsequently, we use machine learning-based methods to identify objective behavioral differences between male and female speakers, identifying the most discriminative features for gender distinction through post-hoc explainability methods.

#### 5.3.1 Expert evaluation of speakers' multimodal nonverbal behaviors

The analysis of the behavioral sub-items assessed by the expert revealed *significant effects of speaker gender and virtual audience social attitude*. However, no significant effect of audience gendered appearance on speakers' behavior was observed. Our results indicate that speakers were more static when facing a positive audience (*median* = 4, *Q*_1_ = 2, *Q*_3_ = 5) than when facing a negative audience (*median* = 3, *Q*_1_ = 2, *Q*_3_ = 4), regardless of their gender, *F*(1, 265) = 4.57, *p* < 0.05.

Regarding participant gender, significant effects were found on the improvement of gesture use during the presentation, *F*(1, 53) = 5.16, *p* < 0.05. These differences were reflected in gesture openness, *F*(1, 53) = 6.78, *p* < 0.05, and body protection behaviors, which indicate whether a participant appears uncomfortable or comfortable based on their overall posture, *F*(1, 53) = 8.69, *p* < 0.01. A significant effect of participant gender was also found on the staticity of spatial displacements, as measured by the maximum displacement amplitude along the x-axis, *F*(1, 53) = 4.57, *p* < 0.05.

Overall, the expert considered that female speakers (*median* = 5, *Q*_1_ = 3, *Q*_3_ = 5) needed greater improvement in their use of gestures during oral presentations than male speakers (*median* = 4, *Q*_1_ = 2, *Q*_3_ = 5). More specifically, male speakers displayed more open gestures (*median* = 3, *Q*_1_ = 1.25, *Q*_3_ = 4) than female speakers (*median* = 2, *Q*_1_ = 1, *Q*_3_ = 3) and appeared more comfortable in terms of body protection behaviors (*median* = 4, *Q*_1_ = 3, *Q*_3_ = 5) than female speakers (*median* = 3, *Q*_1_ = 2, *Q*_3_ = 4). Regarding spatial displacements, female speakers were also more static during their presentations (*median* = 4, *Q*_1_ = 2, *Q*_3_ = 5) than male speakers (*median* = 3, *Q*_1_ = 2, *Q*_3_ = 4). However, it is noteworthy that the expert did not identify any gender differences in verbal behavior.

Although the expert found no differences in overall performance between male and female speakers as indicated in Section 5.2, he identified differences in multimodal nonverbal behavior during the public speaking task according to speaker gender. Nevertheless, these observed behavioral differences may reflect either genuine variations in multimodal nonverbal behavior or subjective perceptions on the part of the expert. To investigate this issue, we conducted an automatic analysis of participant gender based on multimodal nonverbal behavioral features. This analysis allows us to determine more precisely whether speakers' nonverbal behavior differs according to gender and, if so, to identify the specific behavioral features that genuinely distinguish the two groups.

#### 5.3.2 Automatic gender classification based on nonverbal behaviors

To investigate whether nonverbal behaviors differed according to speaker gender, an MLP classifier was trained following the machine learning framework detailed in Section 4.2, using the behavioral features described in Section 3.4.2 to automatically predict the speaker gender. As indicated in Table 2, the model achieved a weighted F1-score of 0.81 and an AUC of 0.86 on the test set, indicating that multimodal nonverbal behavioral cues contain sufficient information to discriminate between male and female speakers. The relatively small difference between training (AUC = 0.91) and test (AUC = 0.86) performances, as indicated by the ROC curve presented in Figure 5, further suggests good generalization capabilities and limited overfitting.

**Table 2:** Overall performance of the MLP model for speaker gender prediction from nonverbal behavioral features.

Model	F1-score	Precision	Recall	AUC
MLP	0.81	0.87	0.73	0.86

**Figure 5:**
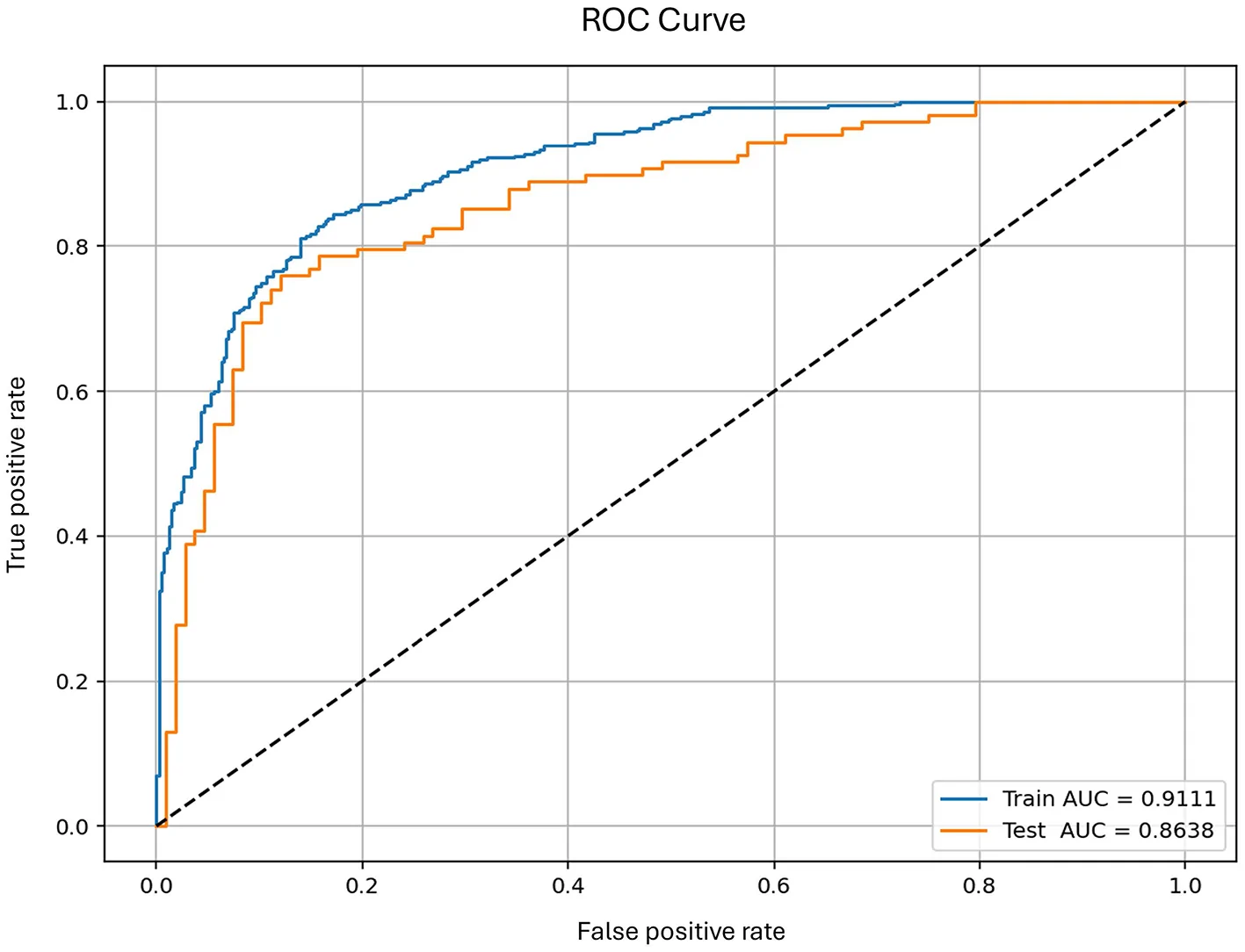
Receiver operating characteristic (ROC) curves for speaker gender prediction obtained with the Multilayer Perceptron (MLP) model on the training and test sets. The curves illustrate the trade-off between the true positive rate (sensitivity) and the false positive rate (1 − specificity) across classification thresholds. The model achieved an Area Under the ROC Curve (AUC) of 0.91 on the training set and 0.86 on the test set. The dashed diagonal line represents the performance of a random classifier (*AUC* = 0.5).

To better understand the behavioral patterns driving these predictions, we applied the SHAP post-hoc explainability method on the learned model as described in Section 4.2. The SHAP analysis is presented in Figure 6. Positive SHAP values drive the prediction toward the female speaker class, whereas negative values orient the prediction toward the male speaker class. Features are ranked in decreasing order of importance. A wider dispersion of SHAP values indicates a stronger impact of the corresponding feature on gender prediction. Moreover, the color of the points further reflects whether the feature value is low or high for a given observation.

**Figure 6:**
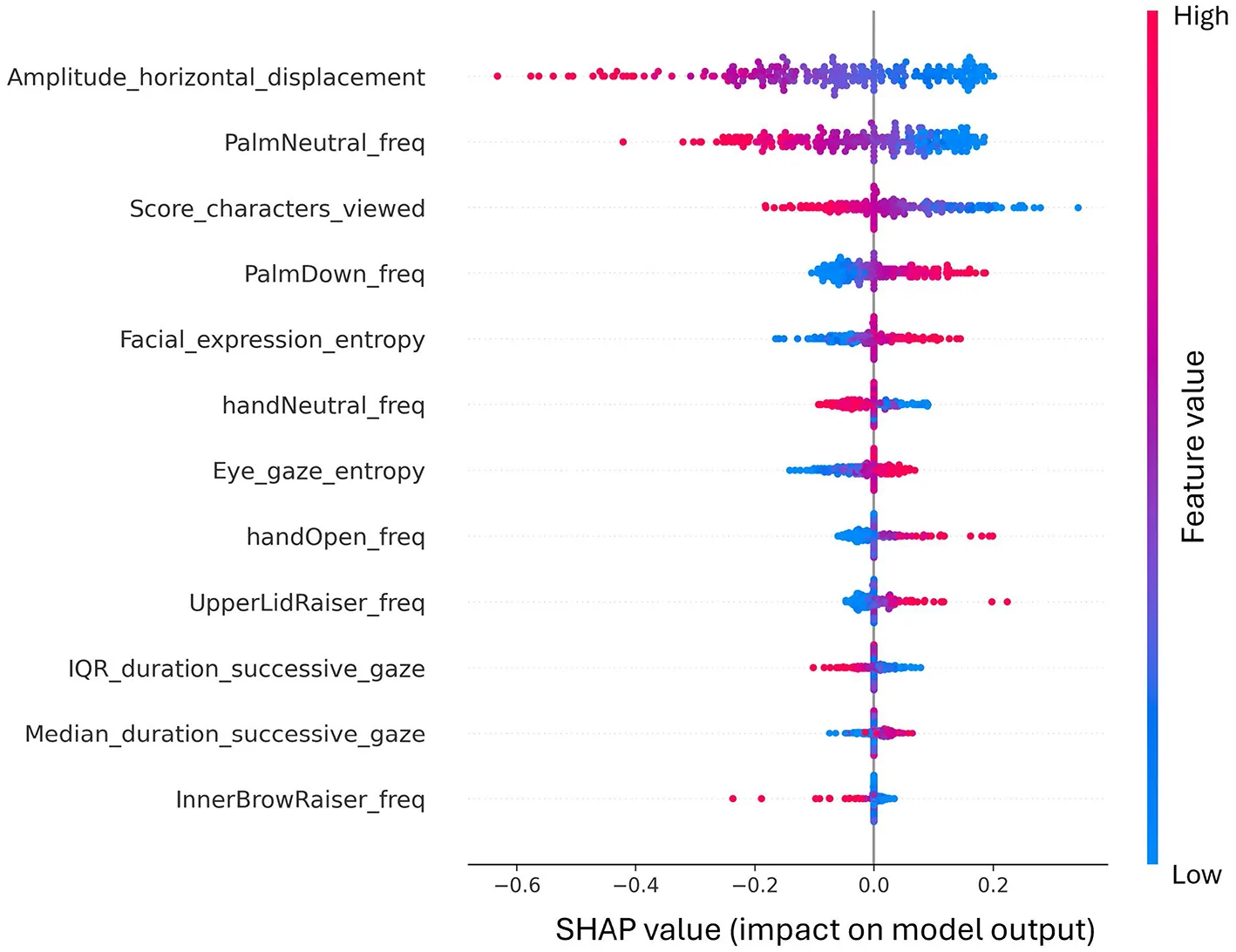
SHAP summary plot showing the contribution of nonverbal behavioral features to speaker gender classification. Positive SHAP values favor the prediction of the female speaker class, whereas negative values favor the male speaker class. Features are ranked according to their overall importance in the model.

Regarding the SHAP analysis, the results suggest that *horizontal displacement amplitude*, which corresponds to the maximum amplitude of speaker displacement along the x-axis, is the feature identified by the model as the most important for predicting speaker gender. Other highly discriminative features included *palm orientation frequencies, gaze behaviors*, and *facial expression* variability. More specifically, female speakers tended to exhibit smaller horizontal displacement amplitudes, used neutral palm orientations less frequently while displaying downward-oriented palms more often, and directed their gaze less uniformly toward the audience. In addition, female speakers showed greater variability in facial expressions, particularly through more frequent upper lid raiser movements, which are commonly associated with emotions such as surprise, fear, or anger. Conversely, male speakers generally displayed larger horizontal displacement amplitudes, more frequent neutral palm orientations, and more balanced gaze behavior, with their attention being more evenly distributed across members of the virtual audience.

Overall, these findings support the expert-based observations by showing that male and female speakers exhibit distinguishable nonverbal behavioral patterns, even though the expert identified no gender differences in overall presentation performance.

## 6 Discussion

This study aimed to examine how speaker gender and the virtual audience's gendered appearance influence public speaking through a multidimensional approach, focusing on subjective experience, performance outcomes, and multimodal behavioral patterns within a VR environment. Specifically, we examined how a virtual audience, varying in social attitude and gendered appearance, affected emotional experience and performance, as measured through self-reported performance and expert-rated performance. Additionally, we analyzed the multimodal behavior of speakers to determine whether gender-based patterns emerge during public speaking. To achieve this, we developed a VR simulation that allowed for the systematic manipulation of audience characteristics while simultaneously capturing a comprehensive dataset of multimodal behavioral data. Our findings reveal that while the virtual audience social attitude significantly influenced the speaker's emotional experience and self-reported performance, no corresponding effect was detected in expert-rated performance. In contrast, the influence of speaker gender was primarily evident in multimodal nonverbal behavior.

### 6.1 Influence of virtual audience characteristics

As expected, our findings indicate that audience social attitude was one of the factors that most strongly influenced participants' experience during public speaking. Specifically, social attitude significantly affected perceived public speaking anxiety, emotional states, and self-evaluations of performance. Participants reported feeling less anxious, experiencing more positive emotions, and rating their own performance more favorably when addressing a positive audience than a negative one. These results suggest that a supportive audience creates a more favorable context for public speaking, positively influencing the subjective emotional experience associated with the task. Given that audience attitude influenced participants' subjective experience, an effect on speaking performance assessed by an external rater might also have been expected, as reported in previous research (Kroczek and Mühlberger, 2023). However, no such effect was observed in the present study. This discrepancy may be related to methodological differences with previous studies, as performance assessment could be influenced by several factors, including the type of audience (e.g., real vs. virtual), the public speaking task, the evaluator, and the assessment method. Therefore, under the specific conditions of the present study, the results show that audience attitude influenced participants' subjective experience, while no corresponding effect was detected in expert-rated speaking performance.

Regarding audience gendered appearance, contrary to our expectations, this factor did not significantly influence participants' subjective experience or performance during the public speaking task. No differences were observed in public speaking anxiety, emotional state, self-reported performance, or expert-rated performance across the different audience gendered appearance conditions. These findings suggest that, within our virtual environment and given the manipulation used, the gender composition of the audience had a limited impact on participants' public speaking experience, as previously reported in the literature (Saufnay and Schyns, 2025). In both the present study and previous research, virtual audience gender was primarily operationalized through the gendered appearance of the virtual characters, without manipulating other potentially relevant gender cues, such as their movements or nonverbal behavior. Several methodological factors may therefore help explain this absence of effect. In our study, the nonverbal behaviors used to animate the virtual characters were derived from motion-capture recordings performed by a single female actor, and their perception was validated only using a female virtual character (Ristorcelli et al., 2024). The same behavioral animations were subsequently applied to all virtual audience members, regardless of their gendered appearance. Therefore, manipulating appearance alone may not be sufficient to produce clearly distinguishable perceptions of virtual characters' gender. Previous work has shown that perceived gender in virtual characters relies on the integration of both appearance and motion cues rather than on appearance alone (McDonnell et al., 2009). More broadly, gender is a social construct that extends beyond physical appearance and also encompasses socially expected behavioral patterns. These methodological choices may have reduced the salience of the audience-gender manipulation in the present study. Moreover, participants' perception of the intended gender differences between the virtual audience members was not explicitly assessed. Consequently, the absence of a detectable effect cannot be distinguished from insufficient salience of the manipulation.

### 6.2 Speaker gender differences in emotional experience and performance

In our study, no significant gender differences were observed in participants' emotional experience or performance during the public speaking task. Male and female speakers did not significantly differ in their levels of public speaking anxiety, emotional state, self-reported performance, or expert-rated performance. Similarly, no significant gender differences were observed at baseline for either public speaking anxiety or emotional state. Thus, across the measures considered in our study, male and female participants reported comparable subjective experiences and achieved comparable levels of overall public speaking performance.

These findings contrast with previous studies reporting gender differences in public speaking anxiety, with female speakers generally reporting higher levels of anxiety or greater fear of being perceived as poor speakers than male speakers (Perveen et al., 2018; Rahman Hz, 2022). Similarly, although previous work has suggested that male speakers may perceive themselves as more competent in oral communication (Alkhutaba, 2022), no gender difference in self-evaluated performance was observed in the present study. Importantly, the absence of gender differences was not restricted to emotional experience, as no significant difference was observed in expert-rated performance either.

Several characteristics of our sample and experimental protocol could have contributed to the absence of significant gender differences. First, our inclusion criteria excluded individuals with high levels of public speaking anxiety, which may have resulted in a sample with a restricted range of anxiety levels. This selection may therefore have limited our ability to detect gender-related differences in public speaking anxiety. Second, participants were given the opportunity to prepare their speeches at home before the experiment. This preparation phase may have reduced the stress associated with the speaking task. Finally, participants were asked to deliver short informative speeches (e.g., introducing themselves or describing a typical day), rather than engaging in more evaluative or persuasive public speaking tasks. Consistent with these characteristics of the protocol, participants reported relatively low levels of public speaking anxiety throughout the experiment, with median anxiety scores remaining below the midpoint of the 5-point scale.

Taken together, these elements suggest that the experimental setting may not have elicited a sufficiently high level of stress for gender-related differences to become detectable. By contrast, more challenging speaking situations requiring speakers to demonstrate confidence, assertiveness, or persuasive abilities may be more likely to activate gender-related stereotypes, as these competencies have traditionally been associated with men (Koenig et al., 2011). The absence of gender differences observed in the present study should therefore be considered in light of the characteristics of both our sample and experimental setting, and may not necessarily generalize to more stressful or socially evaluative public speaking situations.

### 6.3 Nonverbal behavioral markers of gender

Despite the absence of gender differences in overall public speaking performance, expert behavioral evaluations and automatic analyses revealed notable behavioral differences between male and female speakers. According to the expert assessments, female speakers tended to use the available space less extensively, moved less during their presentations, and adopted less open postures. These observations were consistent with our objective behavioral analyses, which showed that female speakers exhibited lower horizontal displacement and a distinct distribution of hand gestures, characterized by a higher frequency of palm-down positions and a reduced occurrence of neutral palm and hand postures compared to men. Beyond these convergences, the automatic analyses identified additional behavioral differences related to gaze patterns and facial expressions that were not captured by the expert. This discrepancy is largely attributable to the experimental constraints. The positioning of the VR headset partially obscured the speakers' faces, making a systematic expert assessment of facial expressions impractical. Furthermore, the expert evaluation interface, which relied on multiple viewing windows, limited the ability to accurately track the temporal dynamics of the participants' gaze direction. Drawing from the computationally extracted behavioral features, male speakers tended to distribute their visual attention more evenly across audience members, whereas female speakers changed their visual targets more frequently and displayed greater variability in their facial expressions. In particular, female speakers more frequently expressed AU5 (Upper Lid Raiser), an action unit commonly associated with negative emotional expressions.

Several of these behavioral differences are consistent with previous findings on gendered nonverbal communication. In particular, male speakers have been shown to adopt more open body postures than female speakers (Newman et al., 2016; Latu et al., 2019), a pattern that was consistently mirrored in our dataset. Likewise, our findings indicating greater facial expressivity and more frequent gaze shifts among female speakers are in agreement with previous observations of gender differences in nonverbal communication (Delbosc et al., 2024). Previous work has also suggested that some facial expressions are interpreted differently depending on the speaker gender. For instance, Singhal et al. (2018) reported that male speakers may benefit from displaying facial expressions associated with sadness, such as AU4 (Brow Lowerer) and AU20 (Lip Stretcher), whereas female speakers tend to receive more positive evaluations when smiling more frequently and avoiding negatively perceived expressions. More broadly, the interpretation of nonverbal behaviors appears to depend on the speaker gender. For example, Levine and Feldman (2002) showed that sustained eye contact was associated with higher likability ratings for female speakers, whereas similarly high levels of eye contact were associated with lower likability ratings for men.

Despite these behavioral differences, no significant gender differences in overall speaking performance were identified, even though nonverbal cues are generally regarded as critical determinants of oratory success (Jim et al., 2025; Ristorcelli and Ochs, 2026). One possible explanation is that comparable levels of speaking performance can be associated with different nonverbal behavioral profiles in male and female speakers. Social norms and gender stereotypes may encourage speakers to adopt behaviors that are perceived as appropriate or desirable for their gender while still enabling them to be evaluated positively (Singhal et al., 2018). Consequently, similar speaking performance may be achieved through different nonverbal behavioral profiles rather than through a single set of optimal communication behaviors. This further suggests that while some gender-specific behavioral patterns may contribute to effective communication in different ways, others, such as differences in spatial occupation, appear to be less directly related to speaking quality (Ristorcelli and Ochs, 2026).

These findings have important implications for the design of automated public speaking assessment and training systems. If comparable levels of performance can be associated with distinct behavioral profiles, a universal evaluation model may be insufficient to accurately assess speakers or provide effective guidance. Accounting for individual behavioral variability may therefore represent an important consideration in the development of automated public speaking training systems.

### 6.4 Limitations and perspectives

Several limitations of the present study should be considered, as they also provide directions for future research. First, the external evaluation of public speaking performance and behavior relied on a single expert. Although this expert had extensive experience in public speaking assessment, this design did not allow the reliability of these subjective judgments to be assessed through inter-rater agreement. Future studies should therefore involve multiple experts with substantial expertise in public speaking and quantify inter-rater agreement. This would provide a more robust evaluation of both overall performance and specific behavioral dimensions and help determine whether the gender-related behavioral differences identified here are consistently perceived across evaluators.

Second, although both verbal and nonverbal dimensions were considered in the expert evaluation, the automatic behavioral analyses conducted in this study focused exclusively on nonverbal features. Consequently, the present findings provide only a partial characterization of the behavioral differences that may exist between male and female speakers. Future research could extend the proposed computational approach to verbal and vocal features, such as speech rate, hesitations, pauses, pitch, or prosodic variations, while accounting for physiological sex-related differences that may influence some vocal features.

Another limitation concerns the characteristics of the sample and experimental protocol, which may have limited the level of public speaking anxiety elicited during the task. The inclusion criteria excluded individuals with high levels of public speaking anxiety, which may have restricted the range of anxiety levels represented in our sample. In addition, participants were able to prepare their presentations at home before the experiment, and the presentations consisted of short informative speeches on relatively familiar topics rather than more challenging or explicitly evaluative speaking tasks. Consistent with these characteristics, anxiety levels remained relatively low throughout the experiment, with median scores below the midpoint of the scale. These conditions may have limited our ability to detect gender-related differences in emotional experience and performance. Future studies could therefore investigate whether such differences emerge under more stressful or socially evaluative conditions, for example by removing prior preparation, using more challenging presentation topics, or requiring participants to persuade or convince the audience. Recruiting participants with a broader range of initial public speaking anxiety levels could also help determine whether gender-related differences are more likely to emerge under such conditions.

A further limitation concerns the manipulation of virtual audience gender. In the present study, audience gender was operationalized through the gendered physical appearance of the virtual characters, whereas their nonverbal behaviors were determined by the social-attitude condition independently of their gender. Future work could design virtual audiences in which gendered appearance is complemented by nonverbal behaviors intended to convey gender-related behavioral patterns. These behavioral models should be validated beforehand to verify both their perceived gender congruence and their compatibility with the intended positive or negative social attitude. A manipulation check should also verify that participants accurately perceive the intended gender of the virtual audience members.

Finally, future research should examine whether the absence of gender differences in emotional experience and performance, as well as the gender-related differences in nonverbal behavior observed in VR, extend to real-life public speaking situations, for instance by comparing virtual and real audiences. These behavioral differences also raise questions for automated public speaking training systems, particularly regarding whether feedback should account for gender-related behavioral profiles. While personalized feedback may better reflect individual communication patterns, it could also reinforce existing gender stereotypes. Future work should therefore investigate the trade-off between personalized and gender-neutral feedback to support effective training while mitigating such biases.

## 7 Conclusion

This study investigated how speaker gender and virtual audience characteristics influence the public speaking experience in VR through a multidimensional approach combining self-reported measures, expert-rated performance, and multimodal behavioral analyses. Our findings indicate that speaker gender had no significant influence on participants' emotional experience or overall public speaking performance. No significant gender differences were observed in public speaking anxiety, emotional state, self-reported performance, or expert-rated performance. In contrast, our findings revealed substantial gender differences in nonverbal behavior. Both expert behavioral evaluations and automatic behavioral analyses consistently revealed distinct behavioral profiles, with additional differences in gaze and facial expressions identified through the automatic analysis of multimodal behavioral features. Regarding the virtual audience, its social attitude influenced participants' emotional experience and self-reported performance, whereas no significant effect of audience gendered appearance was detected.

Overall, this work demonstrates the value of combining self-reported measures, expert evaluations, and objective multimodal behavioral measures to obtain a more comprehensive understanding of public speaking in VR. Our findings show that comparable levels of public speaking performance can coexist with distinct nonverbal behavioral profiles in male and female speakers, highlighting the importance of considering behavioral variability when studying and assessing public speaking.

## Data Availability

The raw data supporting the conclusions of this article will be made available by the authors, without undue reservation.
